# Special Issue: Microfluidic Lab-on-a-Chip Platforms for High-Performance Diagnostics

**DOI:** 10.3390/diagnostics2010001

**Published:** 2012-03-20

**Authors:** Jens Ducrée

**Affiliations:** *Guest Editor*, Biomedical Diagnostics Institute, National Centre of Sensor Research, School of Physical Sciences, Dublin City University, Glasnevin, Dublin 9, Ireland; E-Mail: jens.ducree@dcu.ie; Tel: +353-1-700-7870

The field of microfluidics has seen breath-taking progress since its beginnings in the 1980s and early 1990s. While much of the initial work was a by-product of mainstream micro-electro-mechanical systems (MEMS) and silicon based fabrication schemes, soon a specialized research field developed. Over the last decade a strong, highly interdisciplinary microfluidics community emerged with roots in classical silicon microfabrication as well as chemistry, physics, biotechnology, medicine and various engineering disciplines.

This special issue will emphasize microfluidic lab-on-a-chip platforms which are deemed a key enabler for high-performance future diagnostics. Amongst the techno-scientific advantages which are intrinsic to these miniaturised systems are: laminar flow conditions; enhanced, diffusion-advection controllable mixing and reaction kinetics; low sample and reagent volumes; availability of capillary flow and surface tension related effects; amenability for large-scale combinatorial assays; and scale matching on the micro-to-nano-range for large biomolecules and cells. On a system level, lab-on-a-chip technologies offer user-friendly sample-to-answer automation, single-use cartridges for potentially biohazardous samples, compact footprint, and simplified instrumentation. These features empower use in decentralized point-of-care settings, for instance as portable devices in doctor’s offices, ambulances, patient self-testing at home and global diagnostics. Lab-on-a-chip platforms also bear a high potential to leverage next-generation companion diagnostics for personalized (stratified) medicine.

